# Metformin use and risk of gastric adenocarcinoma in a Swedish population-based cohort study

**DOI:** 10.1038/s41416-019-0598-z

**Published:** 2019-10-08

**Authors:** Jiaojiao Zheng, Shao-Hua Xie, Giola Santoni, Jesper Lagergren

**Affiliations:** 1 Upper Gastrointestinal Surgery, Department of Molecular medicine and Surgery, Karolinska Institutet, Karolinska University Hospital, Stockholm, Sweden; 2 School of Cancer and Pharmaceutical Sciences, King’s College London, London, UK

**Keywords:** Cancer epidemiology, Gastric cancer, Cancer prevention, Epidemiology

## Abstract

**Background:**

Whether or not the use of metformin decreases the risk of gastric adenocarcinoma is unclear.

**Methods:**

This was a population-based cohort study in 2005–2015. Associations between metformin use and gastric non-cardia and cardia adenocarcinomas were examined within two cohorts; a diabetes cohort of participants using anti-diabetes medications, and a matched cohort of common-medication users, where metformin non-users were frequency matched (10:1) with metformin users for sex and age. Multivariable Cox proportional hazard regression analyses provided hazard ratios (HR) and 95% confidence intervals (CI), adjusting for sex, age, calendar year, comorbidity, *Helicobacter pylori* eradication treatment, use of non-steroidal anti-inflammatory drugs or aspirin and use of statins.

**Results:**

During the follow-up for a median of 5.8 years, 892 (0.1%) participants in the diabetes cohort and 6395 (0.1%) participants in the matched cohort of common-medication users developed gastric adenocarcinoma. Metformin users had no significantly decreased risk of gastric non-cardia adenocarcinoma (diabetes cohort: HR 0.93, 95% CI 0.78–1.12; matched cohort: HR 1.30, 95% CI 1.18–1.42) or cardia adenocarcinoma (diabetes cohort: HR 1.49, 95% CI 1.09–2.02; matched cohort: HR 1.58, 95% CI 1.38–1.81) compared with non-users in both cohorts.

**Conclusions:**

This cohort study with <10 years of follow-up suggests metformin use may not prevent gastric adenocarcinoma.

## Background

Gastric cancer, with adenocarcinoma as the predominant (>95%) histological type, is the 6th most common cancer and 3rd leading cause of cancer death worldwide.^1^ The major anatomical subtypes, non-cardia and cardia adenocarcinomas have distinct aetiology. *Helicobacter pylori* (*H. pylori*) infection is the main known risk factor for non-cardia adenocarcinoma, while obesity and gastro-oesophageal reflux disease are the main known risk factors for cardia adenocarcinoma.^2,3^ Both non-cardia and cardia adenocarcinoma have a poor prognosis with a 5-year overall survival rate <30% in most countries, which highlights the need to identify preventive measures for high-risk groups.^4^

Metformin is a first-line oral glucose-lowering medication, widely used in the treatment of type 2 diabetes. Metformin may decrease the risk of some cancer types,^5^ especially cancer of the breast, liver, colon and rectum.^6–8^ Possible anti-carcinogenic mechanisms of metformin include direct activation of the 5′ adenosine monophosphate-activated protein kinase (AMPK) pathway and indirect effects from lowering the blood glucose and insulin levels.^9^ Studies have shown that metformin can inhibit gastric cancer growth in mice,^10^ decrease the invasion and migration of gastric cancer cells^11^ and inhibit the carcinogenic properties of gastric cancer stem cells.^12^ However, evidence from human studies regarding the role of metformin in the prevention of gastric adenocarcinoma is limited, and the results are inconsistent. Most available studies come from Asian populations and do not separately investigate gastric non-cardia and cardia adenocarcinomas.^13^

This study aimed to test the hypothesis that use of metformin decreases the risk of gastric non-cardia and cardia adenocarcinoma in a Western population.

## Methods

### Source cohort

This study was based on a nationwide population-based cohort, entitled the Swedish Prescribed Drugs and Health Cohort (SPREDH). The details of this cohort have been described elsewhere.^14^ In brief, SPREDH includes 8,421,115 Swedish residents with dispensed records of commonly prescribed medications from 1st July 2005 to 31st December 2015. These commonly prescribed medications were sex hormones and modulators of the genital system; drugs for the treatment of diabetes; drugs used against benign prostatic hypertrophy; hormones, hormone antagonists and related agents in antineoplastic and immune-modulating therapy; diuretics; lipid modifying agents; anti-inflammatory agents, analgesics and platelet-aggregation inhibitors; and drugs for peptic ulcer and gastro-oesophageal reflux disease. The participants of SPREDH accounted for ~51% of the entire Swedish population in 2005.^14^ The SPREDH also contains relevant health data of the participants, retrieved from four nationwide health data registries in Sweden: The Prescribed Drug Registry, Cancer Registry, Patient Registry and the Cause of Death Registry.

The Prescribed Drug Registry records individual-level data on all prescribed and dispensed drugs in Sweden since 1st July 2005.^15^ It covers 84% of the total drug sales in Sweden, the remaining part is medications sold over-the-counter or used in hospitals. Every year, ~66% of the Swedish population had at least one record in the Prescribed Drug Registry.^15,16^

The Cancer Registry records data on all cancer diagnoses in Sweden from 1958 onwards. The completeness and positive predictive value of the diagnosis gastric adenocarcinoma are 98 and 96%, respectively.^17^

The Patient Registry contains data on diagnoses and surgical and medical procedures in hospitals since 1987 and from specialist outpatient care since 2001.^18^ The positive predictive value of in-hospital diagnoses is in the range of 85–95%.^19^

The Cause of Death Registry records dates and causes of death of all Swedish residents since 1952. The completeness of both date of death and causes of death data is at least 99%.^20^

The linkages of individuals’ data between these registries were enabled by the 10-digit unique personal identity number assigned to each Swedish resident upon birth or immigration.

### Study design

This was a population-based cohort study from 1st July 2005 to 31st December 2015. All individuals in SPREDH during this study period were potentially eligible for the study. The three exclusion criteria were: (1) previous cancer diagnosis (other than non-melanoma skin cancer) before the cohort entry (identified by the 7th edition of the International Classification of Diseases [ICD-7] codes 140–209, excluding 191 in the Cancer Registry); (2) gastrectomy conducted before the cohort entry (identified by the Nordic Medico-Statistical Committee Classification of Surgical Procedures codes 4411–4420, 4422, 4424–4426, 4429, 4430, 4432, 4434 and 4435 before 1997 and JDC and JDD from 1997 onwards in the Patient Registry); and (3) age below 18 years at the cohort entry.

### Two study cohorts

Two cohorts were identified from the source cohort, a ‘diabetes cohort’ and a ‘matched cohort of common-medication users’.

*The diabetes cohort*. Individuals in SPREDH were included in the diabetes cohort based on their history of using anti-diabetic medications, defined by any dispensed record of anti-diabetic medication(s) (represented by the Anatomical Therapeutic Chemical (ATC) codes A10A and A10B in the Prescribed Drug Registry) during the study period. The exposed group was users of metformin, with or without other anti-diabetic medications, represented by the ATC codes A10BA02, A10BD02, A10BD03, A10BD05, A10BD07, A10BD08, A10BD10, A10BD11, A10BD13, A10BD14, A10BD15, A10BD16, A10BD17, A10BD18, A10BD20 and A10BD22 in the Prescribed Drug Registry. The unexposed group consisted of users of anti-diabetic medication(s) other than metformin, i.e., metformin non-users. The entry date into the diabetes cohort was the first dispensation date of any anti-diabetic medication. To exclude individuals using anti-diabetic medications for other indications than diabetes, we excluded women with diagnoses of gestational diabetes (represented by the Swedish ICD-10 codes O24.4 and O24.9) or polycystic ovarian syndrome (represented by the Swedish ICD-10 code E28.2) in the Patient Registry. However, if a woman with polycystic ovarian syndrome had a first dispensation record of anti-diabetic medication other than metformin, or a later dispensation record of a second anti-diabetic medication, she would be included at the first dispensation date of any anti-diabetic medication(s) other than metformin. In the latter case, the woman was regarded exposed at the cohort entry. The exposure status was allowed to change, i.e., an unexposed participant became exposed at the first purchase of metformin.

*The matched cohort of common-medication users*. This cohort was also derived from SPREDH. The exposed group was metformin users, defined by those who had any dispensed record of metformin in the Prescribed Drug Registry (details above). However, women with any diagnosis of gestational diabetes or polycystic ovarian syndrome were not excluded from the exposed group. The entry date of the metformin users was the first dispensation date of metformin. For each metformin user, ten non-users of metformin were randomly sampled from SPREDH as comparison participants, using frequency matching by age (± 1year) and sex. Thus, the comparison participants entered the cohort on the same date as their matched metformin users. The exposure status was allowed to change also in this cohort.

### Study outcomes

Newly diagnosed gastric adenocarcinomas during the study period were identified from the Cancer Registry by the ICD-7 code 151 combined with the histology code 096 for adenocarcinoma in the WHO/HS/CANC/24.1 classification. Gastric non-cardia adenocarcinoma (ICD-7 code 151, excluding 151.1, and histology code 096) and cardia adenocarcinoma (ICD-7 code 151.1 and histology code 096) were the outcomes. All cohort members were followed up until the occurrence of any gastric adenocarcinoma, death or the end of the study (December 31st, 2015), whichever occurred first.

### Potential confounders

Seven factors were considered as potential confounders, for their possible associations with both metformin use and risk of gastric adenocarcinoma: (1) sex (male or female), (2) age (continuous), (3) calendar year (categorised as 2005, 2006–2010 or 2011–2015), (4) comorbidity (Charlson comorbidity index, categorised as 0, 1 or ≥ 2), (5) *H. pylori* eradication treatment (yes or no), (6) use of non-steroidal anti-inflammatory drugs (NSAIDs) or aspirin (yes or no) and (7) use of statins (yes or no).

Comorbidities (excluding diabetes) diagnosed within 10 years before the cohort entry were searched in the Patient Registry, and were handled using the well-validated Charlson comorbidity index.^21^ While no direct data on *H. pylori* infection were available, a dispensed record of standard *H. pylori* eradication packages (ATC code A02BD in the Prescribed Drug Registry) was used to represent clinically diagnosed *H. pylori* infection. These *H. pylori* eradication packages account for 85% of the *H. pylori* eradication therapy in Sweden.^22^ *H. pylori* eradication treatment was treated as a time-varying variable. Use of NSAIDs or aspirin^23^ and use of statins^24^ were also identified by the ATC codes in the Prescribed Drug Registry (for NSAIDs and aspirin: M01A, N02BA, B01AC06, C10BX01, C10BX02, C10BX04, C10BX05, C10BX06, C10BX08, C10BX12, C07FX02, C07FX03 and C07FX04; for statins: C10AA and C10B) and were categorised as binary variables (yes or no). To be identified as a user of these medications, an individual had to have at least two dispensation records within the first year after cohort entry.

### Statistical analysis

Multivariable Cox proportional hazards regressions were used to estimate the hazard ratios (HR) with 95% confidence intervals (CI) of gastric adenocarcinomas comparing metformin users with non-users in both the diabetes cohort and the matched cohort of common-medication users. The risks of gastric non-cardia and cardia adenocarcinomas were analysed combined and separately in both cohorts. Two models were applied, a crude model (without any adjustment) and a model with adjustment for the seven potential confounders presented and categorised above.

Six sub-group analyses were performed specifically for the risk of gastric non-cardia adenocarcinoma: (1) A dose–response analysis was examined among all users of metformin only. Metformin use was categorised into three levels according to the total defined daily dose (DDD) in the first year after the first known date of dispensation: <175 DDDs, 175–300 DDDs and > 300 DDDs. *P*-value for trend was tested by treating the dose as a continuous variable based on the median values of each category. The remaining five sub-group analyses were performed only in the diabetes cohort; (2) stratified analyses by sex, age (above and below the median) and duration of follow-up (≤3 years, 3–6 years and ≥6 years); (3) sensitivity analyses, excluding individuals with less than 1 year of follow-up; (4) sensitivity analyses comparing non-users with prevalent users of metformin, with prevalent users being those who had at least one dispensation of metformin within the first year after the Prescribed Drug Registry came into use, i.e., between 2005 July 1st and 2006 June 30th; (5) sensitivity analyses restricted to participants with a history of *H. pylori* eradication treatment and (6) sensitivity analyses restricted to participants with diagnoses of type 2 diabetes throughout the follow-up (represented by the Swedish ICD-10 code E11 in the Patient Registry).

The proportional hazard assumption for the Cox regression analyses was tested by evaluating whether the scaled Schoenfeld residuals on time were constant over time. The assumption was met for all analyses. In the diabetes cohort, the timeline in the Cox regression was attained age, while in the matched cohort the timeline was time since entry into the study. A biostatistician (GS) was responsible for the data management and statistical analyses using the statistical software Stata (Release 15, StataCorp, College Station, TX).

## Results

### Participants in the diabetes cohort

The diabetes cohort included 544,130 participants. At the cohort entry, 334,506 (61.5%) of these were metformin users, and 209,624 (38.5%) were non-users of metformin. A total of 72,643 (13.4%) non-users at baseline started using metformin during the follow-up. The number of person-years exposed to metformin was 2,432,273, and the number of person-years not exposed to metformin was 998,884. Compared with non-users, metformin users had less comorbidity, a higher proportion of users of statins, a higher proportion with *H. pylori* eradication therapy and lower overall mortality (Table 1). During the follow-up for a median of 5.8 years (interquartile range 2.3–10.0), 892 (0.1%) participants developed gastric adenocarcinoma.

**Table 1:** Characteristics of the study participants in the diabetes cohort and the matched cohort of common-medication users, numbers (%)

	Diabetes cohort		Matched cohort	
	Metformin users	Non-users^a^	Metformin users	Non-users
Total	334,506 (61.5)	209,624 (38.5)	411,413 (9.1)	4,114,130 (90.9)
*Sex*				
Men	196,721 (58.8)	120,048 (57.3)	239,256 (58.2)	2,392,560 (58.2)
Women	137,785 (41.2)	89,576 (42.7)	172,157 (41.8)	17,211,570 (41.8)
*Age at entry*, mean ± standard deviation	62.1 (± 12.9)	61.4 (± 19.5)	59.0 (± 13.7)	59.0 (± 13.7)
*Calendar year at entry*				
2005	102,834 (30.7)	142,293 (67.9)	127,136 (30.9)	1,271,360 (30.9)
2006–2010	111,199 (33.2)	41,432 (19.7)	146,175 (35.5)	1,461,750 (35.5)
2011–2015	120,473 (36.0)	25,899 (12.4)	138,102 (33.6)	1,381,020 (33.6)
*Charlson comorbidity*^b^				
0	250,635 (74.9)	135,583 (64.7)	303,840 (73.9)	3,265,518 (79.4)
1	59,847 (17.9)	43,510 (20.8)	75,396 (18.3)	618,631 (15.0)
≥2	24,024 (7.2)	30,531 (14.6)	32,177 (7.8)	229,981 (5.6)
*Use of non-steroidal anti-inflammatory drugs or aspirin*^c^				
No	175,589 (52.5)	109,565 (52.3)	247,988 (60.3)	3,048,169 (74.1)
Yes	158,920 (47.5)	100,059 (47.7)	163,425 (39.7)	1,065,961 (25.9)
*Use of statin*^c^				
No	162,671 (48.6)	129,555 (61.8)	22,841 (54.2)	3,377,102 (82.1)
Yes	171,835 (51.4)	80,069 (38.2)	188,572 (45.8)	737,028 (17.9)
*Helicobacter pylori* *eradication*				
No	324,885 (97.1)	205,024 (97.8)	399,781 (97.2)	4,027,949 (97.9)
Yes, before entry	3515 (1.1)	762 (0.4)	4238 (1.0)	33,813 (0.8)
Yes, after entry	6106 (1.8)	3838 (1.8)	7394 (1.8)	52,368 (1.3)
*Death during follow-up*				
No	282,110 (84.3)	133,819 (63.8)	340,543 (82.8)	3,590,316 (87.3)
Yes	52,396 (15.7)	75,805 (36.2)	70,870 (17.2)	523,814 (12.7)

^a^At study entry

^b^In the 10 years before study entry

^c^In the year after study entry

### Participants in the matched cohort of common-medication users

The matched cohort of common-medication users included 4,525,543 participants. Of these, 411,413 (9.1%) were metformin users and 4,114,130 (90.9%) were age-matched and sex-matched non-users of metformin. The number of person-years exposed to metformin was 2,457,824, and the number of person-years not exposed to metformin was 23,912,966. Compared with non-users, metformin users had more comorbidity, higher proportions of users of NSAIDs or aspirin and statins and higher overall mortality, whereas the proportion with *H. pylori* eradication therapy was similar in users and non-users of metformin (Table 1). During the follow-up for a median of 5.8 years (interquartile range 2.8–9.4 years), 6395 (0.1%) participants developed gastric adenocarcinoma.

### Risk of total gastric adenocarcinoma

In the diabetes cohort, metformin use was not associated with any decreased risk of gastric adenocarcinoma (adjusted HR 1.08, 95% CI 0.92–1.26). Similarly, the results of the matched cohort of common-medication users revealed no decreased risk of total gastric adenocarcinoma in metformin users compared with non-users, but rather an increased risk (adjusted HR 1.38, 95% CI 1.26–1.50) (Table 2).

**Table 2:** Risk of total, non-cardia and cardia gastric adenocarcinomas in metformin users compared with non-users in the diabetes cohort and the matched cohort of common-medication users

	Number of cases	Crude HR (95% CI)	Adjusted HR (95% CI)^a^
*Total adenocarcinoma*			
Diabetes cohort			
Non-users	233	Reference	Reference
Metformin users	659	0.99 (0.85–1.15)	1.08 (0.92–1.26)
Matched cohort			
Non-users	5606	Reference	Reference
Metformin users	789	1.37 (1.26–1.48)	1.38 (1.26–1.50)
*Non-cardia adenocarcinoma*			
Diabetes cohort			
Non-users	173	Reference	Reference
Metformin users	421	0.88 (0.73–1.05)	0.93 (0.78–1.12)
Matched cohort			
Non-users	4061	Reference	Reference
Metformin users	533	1.28 (1.16–1.41)	1.30 (1.18–1.42)
*Cardia adenocarcinoma*			
Diabetes cohort			
Non-users	60	Reference	Reference
Metformin users	238	1.30 (0.97–1.74)	1.49 (1.09–2.02)
Matched cohort			
Non-users	1545	Reference	Reference
Metformin users	256	1.61 (1.39–1.86)	1.58 (1.38–1.81)

*HR* hazard ratio, *CI* confidence interval

^a^Adjusted for sex, age, calendar year, use of non-steroidal anti-inflammatory drugs or aspirin, use of statins, Charlson comorbidity index and *Helicobacter pylori* eradication treatment

### Risk of gastric non-cardia adenocarcinoma

In the diabetes cohort, use of metformin did not influence the risk of gastric non-cardia adenocarcinoma (adjusted HR 0.93, 95% CI 0.78–1.12). In the matched cohort of common-medication users, the risk of gastric non-cardia adenocarcinoma was not decreased, but rather increased, among metformin users compared with non-users (adjusted HR 1.30, 95% CI 1.18–1.42) (Table 2).

In the sub-group analyses, no dose–response relation for metformin use and the risk of non-cardia adenocarcinoma was found. Compared with participants who used a low dose (<175 DDD) of metformin, those who used a moderate dose (175–300 DDD) or a high dose (>300 DDD) did not have any reduced risk of gastric non-cardia adenocarcinoma (Table 3). In the stratified analyses of the diabetes cohort, metformin use was not associated with any decreased risk of gastric non-cardia adenocarcinoma in any of the sex or age groups, or in different durations of follow-up (Table 4). In the sensitivity analyses restricted to prevalent metformin users, restricted to participants who were followed up for at least 1 year, restricted to participants who had received *H. pylori* eradication therapy, and restricted to participants with diagnoses of type 2 diabetes, no substantial changes in risk estimates were found (Table 4).

**Table 3:** Dose–response analysis for the risk of gastric non-cardia adenocarcinoma among metformin users

Dosage^a^	Number of cases	Crude HR (95% CI)	Adjusted HR (95% CI)^b^
Less than 175 DDD	99	Reference	Reference
175–300 DDD	122	1.06 (0.81–1.39)	1.01 (0.77–1.32)
More than 300 DDD	147	0.99 (0.77–1.29)	1.02 (0.77–1.34)
*P* for trend		0.887	0.904

*HR* hazard ratio, *CI* confidence interval

^a^Defined as total Defined Daily Dose (DDD) used in the first year after the first dispensation of metformin

^b^Adjusted for sex, age, calendar year, use of non-steroidal anti-inflammatory drugs or aspirin, use of statins, Charlson comorbidity index and *Helicobacter pylori* eradication treatment

**Table 4:** Sub-group analyses of the risk of gastric non-cardia adenocarcinoma in metformin users compared with non-users in the diabetes cohort

	Number of cases	Crude HR (95% CI)	Adjusted HR (95% CI)^a^
*Stratified analyses*			
Sex			
Men			
Non-users	103	Reference	Reference
Metformin users	258	0.91 (0.72–1.15)	0.95 (0.75–1.21)
Women			
Non-users	70	Reference	Reference
Metformin users	163	0.85 (0.64–1.12)	0.90 (0.68–1.20)
Age			
≤60			
Non-users	23	Reference	Reference
Metformin users	65	0.90 (0.56–1.44)	0.94 (0.58–1.51)
>60			
Non-users	150	Reference	Reference
Metformin users	356	0.88 (0.72–1.06)	0.93 (0.76–1.13)
*Follow-up*			
0–3 years			
Non-users	82	Reference	Reference
Metformin users	160	0.90 (0.69–1.18)	1.01 (0.76–1.33)
3–6 years			
Non-users	54	Reference	Reference
Metformin users	129	0.79 (0.58–1.08)	0.84 (0.61–1.16)
≥6 years			
Non-users	37	Reference	Reference
Metformin users	132	1.01 (0.70–1.45)	1.03 (0.71–1.49)
*Sensitivity analysis 1 (*N *=* *355,461)*^b^			
Non-users	173	Reference	Reference
Metformin prevalent users	250	0.92 (0.76–1.11)	0.92 (0.75–1.13)
*Sensitivity analysis 2 (*N *=* *495,822)*^c^			
Non-users	137	Reference	Reference
Metformin users	374	0.91 (0.75–1.11)	0.95 (0.78–1.17)
*Sensitivity analysis 3 (*N *=* *14,221)*^d^			
Non-users	13	Reference	Reference
Metformin users	55	1.11 (0.61–2.02)	1.13 (0.61–2.06)
*Sensitivity analysis 4 (*N *=* *206,769)*^e^			
Non-users	52	Reference	Reference
Metformin users	219	0.92 (0.68–1.25)	0.94 (0.69–1.28)

*HR* hazard ratio, *CI* confidence interval

^a^Adjusted for sex, age, calendar year, use of non-steroidal anti-inflammatory drugs or aspirin, use of statins, Charlson comorbidity index and *Helicobacter pylori* eradication treatment

^b^Restricted to prevalent metformin users as the exposed group

^c^Restricted to participants who were followed up for at least 1 year

^d^Restricted to participants who had received *Helicobacter pylori* eradication therapy

^e^Restricted to participants who had diagnoses of type 2 diabetes only throughout the follow-up

### Risk of gastric cardia adenocarcinoma

Compared with metformin non-users, the risk estimates of gastric cardia adenocarcinoma were not decreased, but rather increased, among metformin users in the diabetes cohort (adjusted HR 1.49, 95% CI 1.09–2.02) and in the matched cohort of common-medication users (adjusted HR 1.58, 95%CI 1.38–1.81) (Table 2).

## Discussion

This study indicates that metformin use does not decrease the risk of gastric non-cardia or cardia adenocarcinoma in a Western population during the follow-up for a median of 6 years.

Strengths of the study include the population-based cohort design, the use of high-quality data with nationwide coverage and the complete follow-up. This study is also the first to analyse gastric non-cardia and cardia adenocarcinoma separately. Several potential confounders were taken into account, including *H. pylori* infection and use of certain medications that may affect the risk of gastric adenocarcinoma. Yet, a limitation of the study was the lack of information on some other potential confounders, particularly obesity. Obesity is associated with an increased risk of gastric cardia adenocarcinoma, and diabetes patients are generally more obese than non-diabetic people.^25^ Besides, diabetes patients using metformin have been reported to have even higher body mass index than diabetes patients treated by other medications.^26^ Thus, the increased risk estimates of gastric cardia adenocarcinoma among metformin users in this study might well be explained by confounding by obesity. However, confounding by obesity should not be a problem in the analyses of gastric non-cardia adenocarcinoma, because obesity is not a risk factor for gastric non-cardia adenocarcinoma.^27,28^ This was the rationale for conducting the post hoc sub-group analyses for gastric non-cardia adenocarcinoma only. Another limitation was the relatively short follow-up (maximum 10 years), because data on drug use before July 2005 (when the Prescribed Drug Registry started) were not available. It is possible that any protective effect of metformin is limited in the very early stage of gastric carcinogenesis, and therefore may be missed by the current study. Thus, the risks of gastric non-cardia and cardia adenocarcinoma in relation to the use of metformin in our study may only reflect the effect of metformin during a limited observation period.

Previous studies examining metformin use in relation to the risk of gastric adenocarcinoma are few, and have provided inconsistent results.^29–32^ All these studies were performed within diabetes populations, comparing metformin users with those either using other anti-diabetic medications or sulfonylurea.^29–36^ Some studies, mostly from Asian populations, have indicated a decreased risk of gastric adenocarcinoma in metformin users, with HRs ranging from 0.83 to 0.45.^29,32,33,36^ A recent cohort study from Hong Kong in diabetes patients who had received *H. pylori* eradication therapy found a reduced risk of gastric adenocarcinoma in metformin users (HR 0.49, 95% CI 0.24–0.98),^33^ which was inconsistent with our results. This study adds to the limited body of literature in Western populations and the lack of any protective effect is supported by two other studies from Western populations, one from the United States and the other from Italy.^31,35^

A novel aspect of this study was the analyses in the matched cohort of common-medication users, besides a diabetes cohort. While no association between metformin use and risk of gastric non-cardia adenocarcinoma was found in the diabetes cohort, it is interesting that an increased risk of gastric non-cardia adenocarcinoma was suggested among metformin users compared with non-users in this matched cohort. This difference indicates a role of diabetes or diabetes-related factors in the aetiology of gastric adenocarcinoma, which highlight a need for research on this topic. However, the existing literature has provided inconclusive evidence regarding the effects of diabetes on the risks of gastric adenocarcinoma and gastric cardia adenocarcinoma.^37–42^

In conclusion, this large and population-based cohort study in Sweden does not support the hypothesis that use of metformin decreases the risk of gastric non-cardia or cardia adenocarcinoma within the initial years of follow-up.

## Data Availability

All the data in this study were retrieved from the Swedish Prescribed Drugs and Health Cohort. The original data are available from the related registries listed above but restrictions apply to the availability of these data, which were used under license for this study and therefore are not publicly available. The data are, however, available through applications to these registries or reasonable request to the corresponding author (S.X.). The codes for the data analysis are archived by the biostatistician (G.S.).
